# Institutional Notes and News

**Published:** 1910-01-08

**Authors:** 


### Institutional Notes and News.

**Derbyshire Royal Infirmary.**

The annual report records the fact that the past year has been a memorable one in the history of the institution, since, with it, the first century of its beneficent work for the relief of the sick and suffering has been completed. Early in the year the President of the Infirmary, his Grace the Duke of Devonshire, called a special meeting of Governors to review the financial position of the infirmary, and to decide what steps should be taken to increase the annual income of the charity. The outcome of this meeting was the issue of a general appeal for funds. Firstly, for new and increased subscriptions to raise the annual income by £3,500; for donations towards defraying the current account debt; and for £5,000 to build an extension to the Nurses' Home. Up to the close of the financial year on September 28, the total sum received and promised in new and increased subscriptions was £703 9s. 6d.; about half of this sum has been paid in and included in the year's accounts, and the remaining subscriptions are promised for the next year. Towards the extinction of the debt on the current account the sum of £2,039 13s. 6d. has been received. For the Building Fund, for which the sum of £5,000 was asked, donations amounting to £1,091 15s. 1d. have been received and a further sum of £130 is promised.

**Birmingham Dental Hospital.**

At the annual general meeting of this institution, which was held last week under the chairmanship of Sir Oliver Lodge, it was reported that the increase in the attendances showed that the hospital was becoming more widely known, and that the benefits derived from the treatment were much appreciated by the poor of the city. For some years now the committee had felt that something should be done to enable patients to obtain artificial teeth without the necessity of wandering round the city in a fruitless search for notes of recommendation. Towards the end of the financial year, therefore, a scheme was put into operation whereby patients might, if they liked, either pay in whole or in part for the artificial teeth, or, if they still desired it, to present subscribers' notes of recommendation. This had proved a boon to many of the patients, as was shown by the fact that during the three months it had been in operation the hospital had received no less than £58 for this work. The work undertaken in connection with the elementary schools had been progressing steadily throughout the year. At Summer Lane it was found that 96 per cent. of the children had defective teeth, and at Bristol Street Schools slightly over 98 per cent. required treatment. At two schools no less than 3,000 children were examined, and practically the whole of these were treated, the total number of operations performed being 11,999. The number of extractions without anæsthetics showed a decrease, whilst those with anæsthetics showed an increase of nearly 4,000, an appalling illustration of the dental conditions prevalent among the people and the wastage of human health and comfort which neglect of dental hygiene involved.

**Queen's Hospital for Children, Hackney.**

A public meeting was held on January 6, 1910, at 2.30 p.m., at the Shoreditch Town Hall, in connection with the appeal made by this institution, his worship the Mayor (Mr. J. E. Harwood, J.P.), presiding. Measures for assisting the hospital were discussed, and the following resolutions were tabled: (1) That this meeting of the residents and ratepayers of Shoreditch, held under the chairmanship of the Mayor, strongly urges upon the public the vital importance to the people of these crowded districts of keeping open all the wards of the Queen's Hospital for Children, and very earnestly appeals to every responsible man and woman to contribute towards the sum required to enable the disastrous step of closing sixty-two of the beds to be avoided. (2) That in view of the serious danger in which the Queen's Hospital for Children stands of being compelled to close sixty-two of its beds for want of funds to carry on its work amongst the children of the poor, this meeting of residents and ratepayers of Shoreditch unanimously undertakes to use every endeavour to secure immediate assistance for the Institution.

**Christmas at University College Hospital.**

Christmas was celebrated at University College Hospital with as much festivity and amusement as the conditions allowed, having regard to the very many serious cases in the hospital. Every effort was made by the matron, the nursing staff, and the resident medical officers to mark the season and to make everybody as bright and happy as possible. On Christmas Day a special dinner was served, consisting of turkeys and Christmas pudding, and the patients were visited by the Highgate Santa Claus Society, members of which personally distributed a wealth of useful gifts. Tea-time was a specially bright hour in the day, for each patient was allowed to have a friend to tea, and this treat, as is only natural, was greatly appreciated. Boxing Day was the red-letter day for the convalescent patients, who were able to enjoy the fun provided for them by the resident medical officers in an amusing entertainment. It was also a red-letter day for the children, who each received a special present from a Christmas-tree, beautifully decorated by the nursing staff. The whole Christmas arrangements were under the personal direction of the matron and resident medical officers, and the cost was entirely defrayed by a voluntary fund administered by the matron.

